# Частота дефицита витамина D у лиц с предиабетом и вновь выявленным сахарным диабетом 2 типа по данным скрининга в г. Андижане и Мархаматском районе Республики Узбекистан

**DOI:** 10.14341/probl13659

**Published:** 2026-07-22

**Authors:** Ш. К. Юсупова, М. С. Нишанова, К. А. Юсупов, Д. С. Абдуразакова, В. М. Мухамедова, Р. Б. Абдулазизхожиева, Х. Х. Чартакова, Ю. Г. Жулиева, М. И. Хамидова, С. Х. Исмоилова, С. Р. Халилова, Х. Х. Супичаков

**Affiliations:** Андижанский государственный медицинский институт Узбекистан; Andijan State Medical Institute Uzbekistan

**Keywords:** витамин D, предиабет, сахарный диабет 2 типа, скрининг, распространенность, возрастные особенности, vitamin D, prediabetes, type 2 diabetes mellitus, screening, prevalence, age­related features

## Abstract

**ОБОСНОВАНИЕ:**

ОБОСНОВАНИЕ. Дефицит витамина D является одной из наиболее распространенных эндокринных проблем, играющих значительную роль в патогенезе нарушений углеводного обмена. Известно, что низкий уровень 25(OH)D ассоциирован с развитием инсулинорезистентности, метаболического синдрома, предиабета и сахарного диабета 2 типа (СД2). Однако данные о распространенности дефицита витамина D среди населения Узбекистана и его возрастных особенностях ограничены.

**ЦЕЛЬ ИССЛЕДОВАНИЯ:**

ЦЕЛЬ ИССЛЕДОВАНИЯ. Изучить частоту дефицита витамина D у лиц с предиабетом и впервые выявленным СД2 по данным скрининга в г. Андижане и Мархаматском районе Республики Узбекистан.

**МАТЕРИАЛЫ И МЕТОДЫ:**

МАТЕРИАЛЫ И МЕТОДЫ. Проведено скрининговое исследование 3400 человек старше 40 лет (1800 — в Мархаматском районе, 1600 — в г. Андижане) с использованием опросника FINDRISC. У 205 лиц с предиабетом, 102 пациентов с впервые выявленным СД2 и 60 человек контрольной группы были определены уровни 25(OH)D, показатели углеводного и липидного обмена, рассчитан индекс HOMA-IR. Диагностика предиабета и СД2 проводилась согласно критериям ВОЗ.

**РЕЗУЛЬТАТЫ:**

РЕЗУЛЬТАТЫ. Установлено, что средний уровень витамина D был достоверно ниже у пациентов с предиабетом и СД2 по сравнению с контрольной группой (p<0,05). Наиболее выраженный дефицит наблюдался у пациентов с СД2, особенно среди женщин и лиц в возрастных категориях 45–59 и 60–74 лет. В г. Андижане дефицит витамина D отмечен у 100% пациентов с СД2, тогда как в Мархаматском районе — у 40,3%.

**ЗАКЛЮЧЕНИЕ:**

ЗАКЛЮЧЕНИЕ. Дефицит витамина D широко распространен среди пациентов с предиабетом и СД2, причем выраженность гиповитаминоза усиливается с возрастом и чаще встречается у женщин. Полученные данные указывают на необходимость рутинного мониторинга уровня 25(OH)D и разработки профилактических мер в группах высокого риска.

## Abstract (en)

**BACKGROUND:**

BACKGROUND. Vitamin D deficiency is one of the most common endocrine disorders and plays a significant role in the pathogenesis of carbohydrate metabolism disturbances. Low serum 25(OH)D levels are associated with the development of insulin resistance, metabolic syndrome, prediabetes, and type 2 diabetes mellitus (T2DM). However, data on the prevalence of vitamin D deficiency and its age-related characteristics in the population of Uzbekistan remain limited.

**OBJECTIVE:**

OBJECTIVE. To investigate the prevalence of vitamin D deficiency in individuals with prediabetes and newly diagnosed T2DM based on screening data from the city of Andijan and the Markhamat district of the Andijan region, Uzbekistan.

**MATERIALS AND METHODS:**

MATERIALS AND METHODS. A total of 3,400 individuals over 40 years of age were screened (1,800 in Markhamat district, 1,600 in Andijan city) using the FINDRISC questionnaire. Serum 25(OH)D levels, carbohydrate and lipid metabolism parameters, and HOMA-IR index were assessed in 205 subjects with prediabetes, 102 patients with newly diagnosed T2DM, and 60 controls. Prediabetes and T2DM were diagnosed according to WHO criteria.

**RESULTS:**

RESULTS. Mean vitamin D levels were significantly lower in patients with prediabetes and T2DM compared to the control group (p<0.05). The most severe deficiency was observed in patients with T2DM, especially among women and individuals aged 45–59 and 60–74 years. In Andijan city, vitamin D deficiency was detected in 100% of T2DM patients, while in Markhamat district the prevalence reached 40.3%.

**CONCLUSIONS:**

CONCLUSIONS. Vitamin D deficiency is highly prevalent among patients with prediabetes and T2DM, with severity increasing with age and more pronounced in women. These findings highlight the need for routine monitoring of serum 25(OH)D and preventive strategies in high-risk groups.

## Обоснование

Дефицит витамина D, который часто встречается у детей и взрослых, вызывает рахит, остеомаляцию и остеопороз. Большинство органов и иммунных клеток имеют рецептор витамина D, а некоторые также обладают способностью метаболизировать 25‑гидроксивитамин D в 1,25-дигидроксивитамин D. 1,25-дигидроксивитамин D является мощным иммуномодулятором, который также усиливает выработку и секрецию нескольких гормонов, включая инсулин [1][2][3][4].

Дефицит витамина D связан с уменьшением высвобождения инсулина, резистентностью к инсулину и сахарным диабетом 2 типа (СД2) в экспериментальных и эпидемиологических исследованиях [5].

Дефицит витамина D связан с метаболическим синдромом и СД2 в эпидемиологических исследованиях. Неясно, является ли эта связь причинно-следственной или обусловлена дополнительными факторами [6][7].

Было проведено много рандомизированных клинических испытаний с витамином D для улучшения гликемического контроля у пациентов с СД2 или замедления прогрессирования от предиабета к СД2. Однако результаты этих испытаний часто были незначительными или клинически не значимыми. Метаанализы подтвердили эти выводы. В настоящее время проводятся крупные двойные слепые испытания с высокими дозами витамина D по сравнению с плацебо с частотой СД2 в качестве результата [3][9].

Витамин D и риск развития диабета 2 типа у людей с предиабетом был изучен в рамках многостранового исследования, опубликованного в 2023 г. Это систематический обзор и метаанализ индивидуальных данных участников из трех рандомизированных клинических испытаний. Витамин D снижал риск диабета на 15% (коэффициент риска 0,85 [ 95% ДИ, 0,75–0,96]) в скорректированных анализах, с 3-летним абсолютным снижением риска на 3,3% (ДИ, 0,6–6,0%). Эффект витамина D не отличался в заранее определенных подгруппах. Среди участников, отнесенных к группе витамина D, у которых средний уровень 25‑гидроксивитамина D в сыворотке крови поддерживался на уровне не менее 125 нмоль/л (≥50 нг/мл) по сравнению с 50–74 нмоль/л (20–29 нг/мл) в течение периода наблюдения, холекальциферол снизил риск развития диабета на 76% (коэффициент риска 0,24 [ ДИ от 0,16 до 0,36]), с абсолютным снижением риска за 3 года на 18,1% (ДИ от 11,7% до 24,6%) [1][2].

По данным метаанализа трех рандомизированных плацебо-контролируемых исследований с участием 4190 человек, у людей с предиабетом, получавших терапию витамином D, риск развития диабета 2 типа был на 15% ниже в скорректированных анализах, а абсолютное снижение риска составило 3,3% за 3 года, отметил автор [8][10].

Вышеуказанное стало основой для данного исследования.

Цель исследования — изучить частоту дефицита витамина D у лиц с предиабетом и вновь выявленным СД2 по данным скрининга в г. Андижане и Мархаматском районе Республики Узбекистан.

## МАТЕРИАЛЫ И МЕТОДЫ

## Место и время проведения исследования

Место проведения. Обследование было проведено у 1800 лиц Мархаматского района Андижанской области и у 1600 лиц г. Андижана, представляющую собой 10%-ную репрезентативную выборку.

Время исследования. Исследование проводилось в 2023–2024 гг. Забор крови для определения уровня витамина D проводился в осенний период (сентябрь–октябрь).

Критерии включения: мужчины, женщины, возраст — старше 40 лет.

Критерии исключения: дети, подростки, пациенты сахарным диабетом 1 и 2 типов в анамнезе, беременные женщины, пациенты с хроническими заболеваниями печени, почек, желудочно-кишечного тракта.

## Способ формирования выборки из изучаемой популяции (или нескольких выборок из нескольких изучаемых популяций)

Наше исследование включало рандомизацию групп методом случайной выборки и дальнейшего распределения по подгруппам (стратифицированная рандомизация).

## Дизайн исследования

Исследование проводилось в 3 этапа на базе центральной многопрофильной поликлиники Мархаматского района и 8 поликлиники города Андижана.

I этап. На 1 этапе исследования был проведён скрининг населения у 3400 человек с помощью анкеты – опросника FINDRISC, для выявления факторов риска предиабета и СД 2 типа. Все исследования были проведены с согласия участников.

II этап. На 2 этапе работы проведена выборка людей с высоким риском развития предиабета и сахарного диабета 2 типа. В сформировавшейся группе с высоким риском развития нарушения углеводного обмена была проведена оценка углеводного статуса: глюкоза натощак, гликированный гемоглобин

Диагноз предиабет устанавливался в соответствии с критериями, предложенными ВОЗ.

III этап. На 3 этапе у 205 лиц с предиабетом, 102 лиц с вновь выявленным сахарным диабетом и у 60 лиц без нарушения углеводного обмена были изучены уровни витамина 25(ОН)D3, инсулин, липидного спектра в крови, рассчитан индекс инсулинорезистентности (HOMA-IR).

## Методы

Нами были выполнены биохимические и гормональные исследования крови. Параметры углеводного обмена оценивались на основании оценки уровней гликемии капиллярной крови с помощью глюкометров. Реагенты использовались следующие: инсулин — Вектор Бест (Россия), общий холестерин — Хьюман (Германия), ЛПВП — Хьюман (Германия), ЛПНП — Хьюман (Германия), триглицериды — Cypress Diagnostics, Бельгия, гликогемоглобин — Хьюман (Германия), кальций — Хьюман (Германия), Цинк- фирма «DAC-SpectroMed», Витамин D — Diagnostics Biochem Canada Inc.» [ 25‑гидроксивитамин D или 25(ОН)D] с помощью полуавтоматического биохимического анализатора Mindray BA-88A). Дефицит витамина D определялся по международным рекомендациям Endocrine Society 2011 года- тяжелый дефицит менее 10 нг/мл, дефицит менее 20 нг/мл. Недостаточность витамина D определялась как Dот 20 до 30 нг/мл, норма как более 30 нг/мл. [2].

## Статистический анализ

Для статистической обработки данных использовались методы описательной статистики, включая расчёт среднего значения и стандартного отклонения уровня витамина D для каждой группы. Для анализа различий между группами с учетом региона (Мархамат и Город) применялся t-тест для независимых выборок, с предварительным тестированием нормальности распределения с использованием теста Шапиро-Уилка. Статистическая значимость различий была установлена на уровне p<0,05. Все графические данные представлены в виде столбчатых диаграмм, отображающих средние значения уровня витамина D среди различных групп.

## Этическая экспертиза

Протокол исследования одобрен локальным этическим комитетом МЗ РУз протокол № 7/18-2129 от 26.06.2023

## Результаты

Исходя из цели исследования получены следующие данные: в Мархаматском районе предиабет был выявлен у 102 (5,6%) пациентов из 1800 обследованных, а в г. Андижане — у 103 (6,4%) из 1600 обследованных жителей групп риска. Из них в Мархаматском районе и г. Андижане — нарушение гликемии натощак (НГН) — (19/20), нарушение толерантности к глюкозе (НТГ) (30/30), НГН + НГН (53/50), сахарный диабет 2 типа (СД2) (52/50) и без нарушений углеводного обмена (без НУО) — (30/30).

На рисунках 1, 2 представлены средние концентрации витамина D (нг/мл) в различных группах пациентов. На рисунке 1 показано, что в группе с нарушенной гликемией натощак (НГН) зафиксирован наименьший уровень витамина D — 12,8±1,0 нг/мл. В группе с нарушенной толерантностью к глюкозе (НТГ) уровень витамина D был выше — 16,1±1,0 нг/мл, и статистически значимо превышал показатель группы НГН (p<0,05). У пациентов с сочетанием НГН и НТГ средний уровень витамина D составил 14,5±0,75 нг/мл; этот уровень статистически значимо выше (p<0,05). В группе больных СД2 средний уровень витамина D оказался 11,8±0,87 нг/мл.

**Figure:**
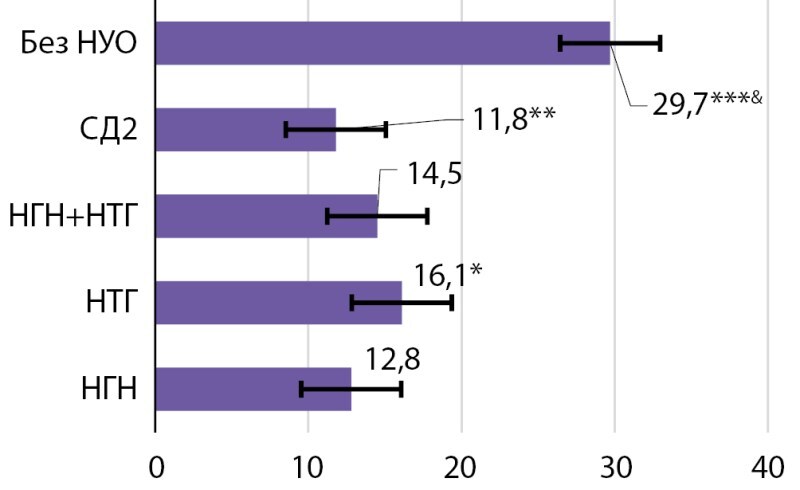
Рисунок 1. Средний уровень витамина D по группам в Мархаматском районе. Примечание: * — достоверно по сравнению с показателями НГН группы (* — P<0,05), достоверно по сравнению с показателями НТГ группы; **<0,01; ***<0,001); & — достоверно по сравнению с показателями НГН+НТГ группы (& — P<0,001).

**Figure:**
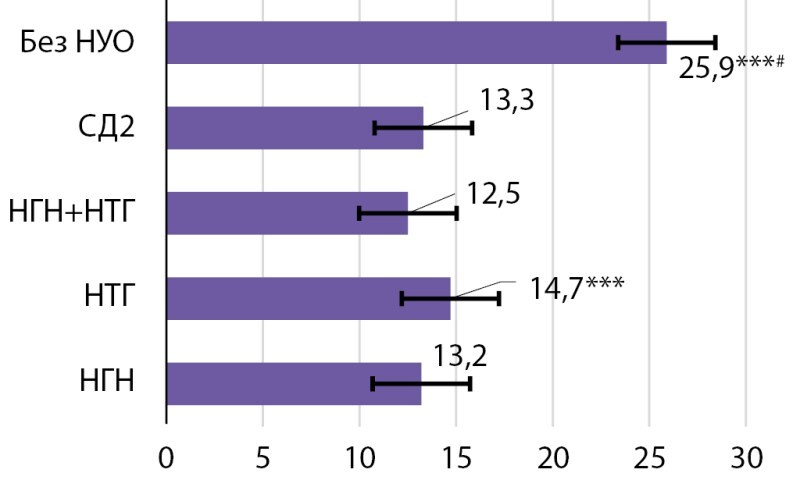
Рисунок 2. Средний уровень витамина D у жителей г. Андижана (нг/мл). Примечание: * — достоверно по сравнению с показателями НГН группы (* — P<0,05 ***P<0,001); # — достоверно по сравнению с показателями СД группы (# — P<0,05).

Результаты показывают, что пациенты с нарушениями углеводного обмена (НГН, НТГ и СД 2) имеют значительно более низкие уровни витамина D по сравнению с контрольной группой, причем наименьшие показатели были зарегистрированы в группе с СД2. Аналогичный анализ по распределению степени дефицита витамина D в клинических группах исследования был выполнен среди пациентов г. Андижана (рис. 2). Из рисунка 2 видно, наибольший уровень витамина D зарегистрирован в контрольной группе без нарушения углеводного обмена (25,9±1,3 нг/мл), статистическое значение относительно групп с нарушениями углеводного обмена (p<0,001). В группе с НГН уровень витамина D составил 13,2±0,77 нг/мл, в группе с НТГ — 14,7±1,0 нг/мл, а в группе с сочетанием НГН и НТГ — 12,5±0,5 нг/мл. Уровень витамина D в группе СД2 составил 13,3±0,79 нг/мл. Наименьший уровень витамина D наблюдается в группе НГН+НТГ.

Далее нами было изучено среднее значение витамина D у женщин и мужчин в зависимости от региона (рис. 3 и 4).

**Figure:**
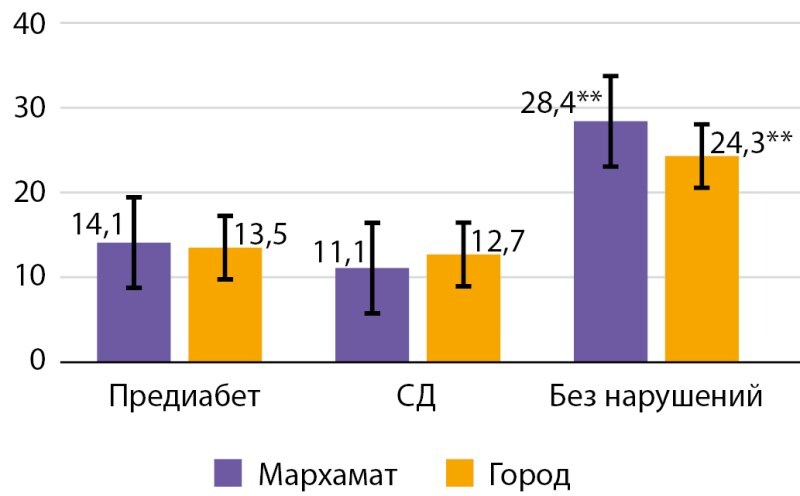
Рисунок 3. Среднее значение витамина D (нг/мл) у женщин в зависимости от региона. Примечание: * — достоверно по сравнению с показателями группы «предиабет, СД» (** — P<0,001).

**Figure:**
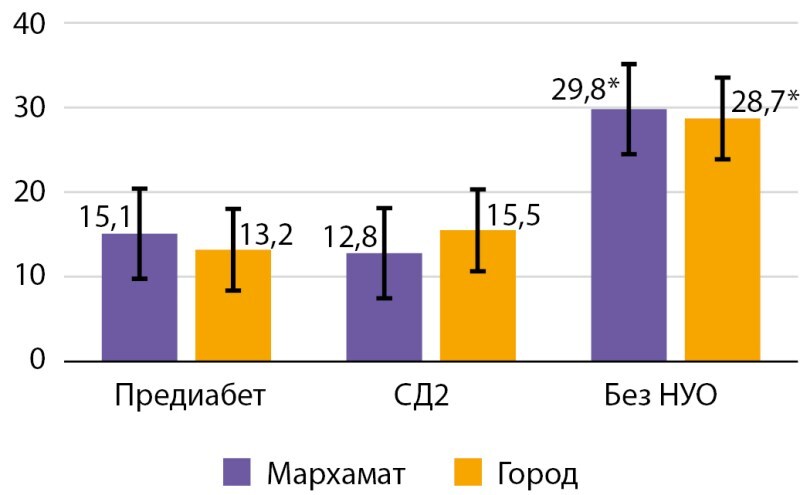
Рисунок 4. Среднее значение витамина D (нг/мл) у мужчин в зависимости от региона. Примечание: * — достоверно по сравнению с показателями группы «предиабет, СД» (* — P<0,001).

На рисунке 3, 4 показано среднее значение витамина D у женщин и мужчин в зависимости от региона, из рисунка 3 видно, что у женщин с предиабетом уровень витамина D был ниже в 1,8–2,0 раза, а при СД2 — в 1,9–2,6 раза по сравнению с контрольной группой. Наименьшие значения зафиксированы у женщин с СД2 из Мархаматского района (11,1 нг/мл против 28,4 нг/мл в контроле). У мужчин (рис. 4) с предиабетом уровень витамина D снизился в 2,0–2,2 раза, а при СД2 — в 1,9–2,3 раза по сравнению с мужчинами без нарушений углеводного обмена.

Далее мы изучили распределение степени дефицита витамина D по возрасту в исследуемых регионах (табл. 1). В таблице 1 представлено распределение степени дефицита витамина D по возрастным группам среди пациентов с предиабетом, СД2 и контрольной группой (без нарушения углеводного обмена, НУО) в Мархаматском районе и г. Андижан. В группе «предиабет» Мархаматского района наибольшая распространенность дефицита витамина D наблюдается в возрастной группе 60–74 лет (29,4%), в то время как в группе 18–44 лет преобладает легкий дефицит (18,6%). В группе «СД» наиболее выраженный дефицит витамина D выявлен в возрастных категориях 45–59 лет (25,0%) и 60–74 лет (25,0%). В контрольной группе наблюдается более высокая частота недостаточности витамина D, особенно в возрастных группах 45–59 лет (23,3%) и 60–74 лет (26,7%).

**Table:** Таблица 1. Распределение степени дефицита витамина D по возрасту в исследуемых регионах

Группа	Норма≥30	Недостаточность20–30	Дефицит10–20	Тяжелый дефицит<10
Мархамат	г. Андижан	Мархамат	г. Андижан	Мархамат	г. Андижан	Мархамат	г. Андижан
абс	%	абс	%	абс	%	абс	%	абс	%	абс	%	абс	%	абс	%
Предиабет 102/100	18–44 лет	-	-	-	-	4	3,9	1	0,97	19	18,6	15	14,6	6	5,9	3	2,9
45–59 лет	-	-	1	0,97	5	4,9	-	-	22	21,6	42	40,8	3	2,9	11	10,7
60–74 года	-	-	-	-	5	4,9	5	4,85	30	29,4	22	21,4	8	7,8	3	2,9
СД 52/50	18–44 лет	-	-	-	-	-	-	-	-	2	3,8	3	6	3	5,8	2	4
45–59 лет	-	-	-	-	5	9,6	4	8	7	13,5	18	36	13	25	10	20
60–74 года	-	-	-	-	4	7,7	4	8	13	25	8	16	5	9,6	1	2
Без НУО 30/30	18–44 лет	2	6,7	1	3,3	6	20	5	16,7	-	-	-	-	-	-	-	-
45–59 лет	5	16,7	6	20	7	23,3	13	43,3	-	-	-	-	-	-	-	-
60–74 года	2	6,7	2	6,7	8	26,7	3	10	-	-	-	-	-	-	-	-

Таким образом, среди пациентов с СД2 в Мархаматском районе отмечается значительный дефицит витамина D, особенно в возрасте 45–59 лет и 60–74 года, в то время как в контрольной группе преобладает недостаточность витамина D.

В г. Андижан в группе «предиабет» наибольшее количество людей с дефицитом витамина D наблюдается в возрастной категории 45–59 лет (40,8%), а в группе 18–44 лет преобладает дефицит (14,6%). В группе «СД» также наибольший дефицит витамина D зафиксирован в возрасте 45–59 лет (36,0%) и 60–74 года (16,0%). В контрольной группе частота дефицита витамина D выше в возрасте 45–59 лет (43,3%), в то время как в группе 18–44 лет более выражена недостаточность витамина D (16,7%).

Среди пациентов с СД2 и предиабетом в г. Андижан наибольший дефицит витамина D наблюдается в возрастной группе 45–59 лет, в то время как в контрольной группе преобладает недостаточность витамина D.

## Обсуждение

Результаты исследования показали высокую частоту дефицита витамина D среди пациентов с нарушениями углеводного обмена, включая предиабет и впервые выявленный СД2, причем наиболее выраженный дефицит наблюдался у пациентов с СД2. Полученные данные согласуются с результатами систематических обзоров и метаанализов, в которых сообщается, что распространенность дефицита витамина D у больных СД2 достигает 60–65% и выше [5][6]. Полученные нами результаты также подтверждают наличие более низких значений D в группах с нарушенной гликемией натощак и нарушенной толерантностью к глюкозе по сравнению с контрольной группой, что сопоставимо с данными метаанализа Pittas и соавт. (2023), где было показано, что поддержание адекватного уровня витамина D у пациентов с предиабетом снижает риск прогрессирования в СД2 [1].

Особое внимание заслуживает выявленная зависимость степени дефицита витамина D от возраста. В обеих исследованных популяциях наиболее значимый дефицит регистрировался в возрастных категориях 45–59 и 60–74 лет, что согласуется с литературными данными о снижении кожного синтеза витамина D у лиц старших возрастных групп, уменьшении времени пребывания на солнце, сопутствующей полиморбидности и частом наличии ожирения [4][6]. Аналогичные результаты получены Lips и соавт. (2017), которые продемонстрировали, что возраст является значимым предиктором гиповитаминоза D у пациентов с метаболическими нарушениями [5].

Гендерные различия, выявленные в ходе исследования, также заслуживают внимания: у женщин снижение уровня витамина D было более выраженным, чем у мужчин. Эти данные находят отражение в исследованиях Holick (2008) и других авторов, указывающих на более высокую частоту гиповитаминоза D у женщин, что может быть связано как с особенностями гормонального статуса, так и с образом жизни [4].

Сравнение полученных результатов с данными международных исследований свидетельствует о наличии определенной гетерогенности. Она обусловлена различиями в методах определения уровня витамина D, используемыми пороговыми значениями для диагностики дефицита и недостаточности, климато-географическими особенностями регионов, а также различиями в образе жизни обследованных популяций [2][6][7]. В то же время единым остается факт более низких концентраций витамина D у лиц с нарушениями углеводного обмена по сравнению с контрольными группами.

С патофизиологической точки зрения полученные результаты могут быть объяснены влиянием витамина D на чувствительность тканей к инсулину и секрецию инсулина β-клетками поджелудочной железы. Низкие концентрации витамина D ассоциированы с развитием инсулинорезистентности, нарушением функциональной активности β-клеток и повышением уровня гликированного гемоглобина (HbA1c) [5][7]. В литературе обсуждается также роль витамина D в регуляции системного воспаления, что дополнительно связывает его дефицит с метаболическим синдромом и прогрессированием предиабета в СД2 [6][8]. Наши данные сопоставимы с результатами исследований Kawahara и соавт. (2022), которые показали положительный эффект терапии активными формами витамина D на замедление прогрессирования предиабета в СД2 в японской популяции [8].

Полученные результаты имеют важное клиническое значение. Высокая распространенность дефицита витамина D среди лиц с предиабетом и СД2, особенно в возрастных группах старше 45 лет, обосновывает необходимость регулярного контроля уровня D в крови в этой категории пациентов. Выявление и своевременная коррекция дефицита могут рассматриваться как один из компонентов комплексной профилактики прогрессирования нарушений углеводного обмена и снижения риска сердечно-сосудистых осложнений.

Таким образом, результаты исследования подтверждают тесную взаимосвязь дефицита витамина D с нарушениями углеводного обмена и подчеркивают значимость учёта возрастных и гендерных факторов при оценке риска и разработке профилактических мероприятий.

## Заключение

У пациентов с предиабетом и впервые выявленным СД2 зарегистрированы статистически значимо более низкие уровни витамина D по сравнению с контрольной группой без нарушений углеводного обмена. Наиболее выраженный дефицит выявлен в группах с СД2, преимущественно среди женщин и лиц в возрастных категориях 45–59 и 60–74 лет. В г. Андижане дефицит витамина D отмечался у всех обследованных пациентов с СД2, тогда как в Мархаматском районе его частота составила 40,3%.

## Дополнительная информация

Источники финансирования. Отсутствуют.

Конфликт интересов. Авторы декларируют отсутствие явных и потенциальных конфликтов интересов, связанных с содержанием настоящей статьи.

Участие авторов. Все авторы внесли значимый вклад в проведение исследования и подготовку статьи, прочли и одобрили финальную версию статьи перед публикацией, а также согласились нести ответственность за все аспекты работы, гарантируя надлежащее рассмотрение и решение вопросов.
